# Hair Whorl Patterns Relating to Equine Behavior and Laterality in Hungarian Thoroughbred Racehorses

**DOI:** 10.3390/vetsci13030289

**Published:** 2026-03-19

**Authors:** Attila Zsolnai, Judit Kis, Boglárka Czinege, László Rózsa, Péter Póti, Ferenc Husvéth, István Anton

**Affiliations:** 1 Institute of Animal Sciences, Hungarian University of Agriculture and Life Sciences, Guba Sándor utca 40, 7400 Kaposvar, Hungary; 2 Institute of Animal Sciences, Hungarian University of Agriculture and Life Sciences, Deák Ferenc utca 16, 8360 Keszthely, Hungary; kis.judit@uni-mate.hu (J.K.); czinege.boglarka07@gmail.com (B.C.); rozsa.laszlo@uni-mate.hu (L.R.); anton.istvan@uni-mate.hu (I.A.); 3 Institute of Animal Sciences, Hungarian University of Agriculture and Life Sciences, Páter Károly utca 1, 2100 Godollo, Hungary; poti.peter@uni-mate.hu; 4 Institute of Physiology and Nutrition, Hungarian University of Agriculture and Life Sciences, Deák Ferenc utca 16, 8360 Keszthely, Hungary; husveth.ferenc@uni-mate.hu

**Keywords:** ethology, Trichoglyphs, sidedness, *Equus caballus*, temperament

## Abstract

Hair whorl patterns are usually used as a predictor of equine laterality, temperament and reactions to various environmental stimuli. Thus, temperament and motor laterality may be deducted in order to ease the daily work, training, handling and transportation of Thoroughbreds. We aimed to identify the number, position and orientation of hair whorl patterns to find the association between environment stimuli and the laterality, affability, trainability and anxiety of 81 individuals. We analyzed hair whorls with SPSS (Statistical Package for the Social Sciences) accompanied by a laterality paddock test and temperament questionnaire. All of our examined Thoroughbreds showed at least one circular whorl on their head. Mostly, the whorls were located on the top of the head. Compared to previous studies, the presence of linear whorls on the body was also high. We found that the counterclockwise head and clockwise whorls were associated with left and right front leg preference (*p* < 0.001).

## Simple Summary

This study explored the relationship between the number, position, and direction of hair whorls across the entire body of horses and their temperament traits (affability, trainability, anxiety) and front leg preference. It found that the direction of head whorls was linked to front leg preference during a paddock test. Furthermore, the total number of hair whorls on a horse correlated positively with scores for affability and trainability. These findings mean that observing hair whorl patterns, particularly their location, direction, and total count, might offer insights into a horse’s laterality and disposition regarding friendliness and ease of training. Such observations could potentially help predict behavior, aiding in management, training, and handling.

## 1. Introduction

Ever since the 18th century, the Thoroughbred studbook has been closed and the breed is mainly used for racing purposes. During the 300-year-old breeding history, the main selection criteria was speed [1]. Therefore, the phenotype of present-day racehorses is exceedingly homogeneous. The condition for studbook registry of Thoroughbreds is the closed bloodline which means that all ancestors must be Thoroughbreds. It can be noted that Thoroughbreds tend to be more reactive to environment stimuli than half-breds [2,3].

This reactivity has led to investigating external physical markers that might correspond to a horse’s underlying neurological profile. Such physical markers could be hair whorls developing from the same embryonic layer as the nervous system [4].

Hair whorls are coat patterns that may appear in different shapes. They can be circular or linear swirls (feather-like shape). In different hair whorls, horizontal and vertical arrangement are differentiated. Rotational direction can be clockwise, counterclockwise or radial. It was already documented in the late 20th century that whorl patterns, position and behavioral traits are associated [5,6].

Studies confirmed an association between whorls and sperm morphology in cattle [7]. In dogs, such coat patterns relate to the length and thickness of hair, sex [8,9], motor and sensorial laterality [9,10]. In humans, parietal whorl patterns on the head are associated with disorders of brain development [11]. Certain researchers conclude that right or left handedness, language lateralization and whorl development are explained by the same genetic mechanism [12,13]. Despite these findings, other researchers dispute these statements [14,15].

The genetic background of the hair whorls is not entirely clarified; however, it is widely recognized that the heritability (h^2^) of hair whorls is high, usually between 0.66 and 0.99 in horses [16,17]. These estimates show that hair whorl patterns have high additive genetic variance, underlining this phenotypic trait as a good indicator of genotype. Studies on Polish Konik horses [17] also reported associations between hair whorls on the head and behavior like temperament traits and response to novel objects. Randle et al. [18] and Encina et al. [16] also found correlations between hair whorl patterns and temperament; the latter suggests the consideration of the location of these patterns in the breeding plan of Pura Raza Española (PRE) horses.

Murphy and Arkins [19] identified an association between the hair whorl rotations of the head and the motor laterality, e.g., an individual with a clockwise whorl on the head tends to be right-handed. The genetic background of the development of motor laterality is unresolved; however, its recognition and implementation during the training process may be pivotal [20]. Choosing the right training method might be crucial especially for young horses, since the wrong choice may intensify lateral asymmetry [21]. Observations like body asymmetry, motor and sensory laterality help to predict the anticipated reaction and consequently offers advances in the training procedure [22]. Handedness is influenced by the association between motor laterality and hair whorls [19]; hence, there is bias to performance during training and racing [23,24].

The aim of this study was to test for the possible associations between the number, rotation and position of hair whorls found on the whole body, and temperament in Thoroughbred racehorses, which may influence motor laterality. All the above-mentioned traits may have an impact on race performance and success. The different temperaments of athletic horses can also have a significant influence on management and training schedules.

## 2. Materials and Methods

### 2.1. Database of Physical Traits

We collected data on 81 Hungarian Thoroughbred racehorses; median age of animals was 3 years (range: 2–13), 5 were geldings, 28 were mares and 48 stallions. As for the color, 49 were bay, 14 chestnut, 1 black and 17 were of gray color. All of the investigated Thoroughbreds were active racehorses in training from 2 to 13 years of age (*n*_2_ = 24, *n*_3_ = 31, *n*_4_ = 9, *n*_5_ = 5, *n*_6_ = 2, *n*_7_ = 1, *n*_8_ = 2, *n*_9_ = 1, *n*_11_= 1, *n*_12_ = 5). They were all trained and stabled at the same training center (Alagi Versenyló Tréningközpont, Dunakeszi, Hungary), under the same stable and training conditions for competitive racing purposes, with the same amount of daily workload. In our study, circular and linear hair whorls were primarily identified and counted on main body parts (head, neck, body, flanks and limbs). Hair whorls on the head were counted from the lips to the bottom line of the ears. Horses were categorized as having radial, counterclockwise, or clockwise whorls based on the patterns of their hair whorls on the head.

Positions of the hair whorls on the head were divided into the following three main group categories and subcategories [18]:

- Below eye level (low right, low center, low left);
- Between the eyes;
- Above eye level (top right, top center, top left).

**Figure 1:**
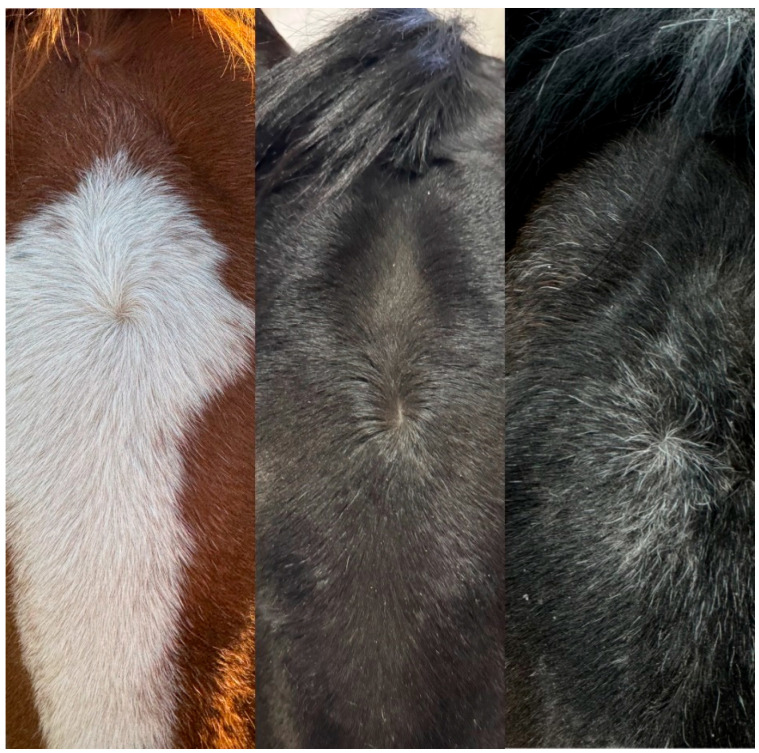
Hair whorl types: clockwise (**left**), radial (**middle**) and counterclockwise (**right**).

On the neck (both left and right side), whorls were counted from atlas vertebra (C1) to scapulae; these parts also include the chest area (central). Furthermore, the symmetry of the whorls was examined all over the body, focused on body parts mentioned above. We adopted the same type of blank charts that are used in the horse passports to record hair whorl positions. The orientation of the circular hair whorls was noted. If a horse had only clockwise and radial–circular head whorls (and did not have any counterclockwise hair whorls), we categorized them as clockwise orientation preference. Similarly, if the hair whorls on the head were either all counterclockwise or counterclockwise and radial (and no clockwise hair whorls were present), we categorized them as counterclockwise direction preference. If both clockwise and counterclockwise hair whorls were present or all hair whorls were radial on the head, we categorized the horse as having ambidextrous direction preference with regard to the head hair whorls (Figure 1).

### 2.2. Paddock Experiment

**Figure 2:**
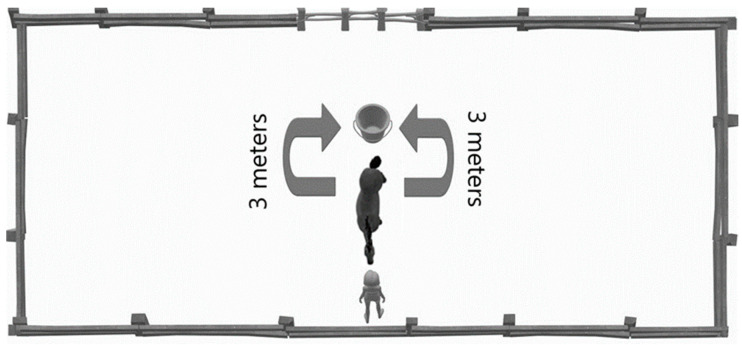
Experimental setup of paddock test. Horse is led to the depicted position. The handler and the horse face each other; the food bucket is behind the horse.

All horses were examined at the same 16 × 21 m rectangular, open-sided, sandy paddock (they were able to see the surroundings) without the possibility of grazing. Each horse went to the paddock with varying regularity. Observations (concerning turning and leg preferences) took place on consecutive days, between 8:00 a.m. and 12:00 p.m., with no other horses around. The horses were fed at 6 a.m. daily. The handler, always the same person, got acquainted with the individual horses in advance and wore plain black clothing during the experiment. Following a ~20 min observation period outside the paddock, where the horses accustomed to the area, the handler brought an individual inside. Left alone and free within, it could release excess energy and become familiar with its surroundings. After calming down in the completely quiet and undisturbed environment and standing still for 5 min, a small amount of sliced carrot (50 g) was placed in a 20-liter plastic container in a central position. The container, a bucket, was consistently placed in the same location for all subjects. The caregiver was instructed to carry and place the bucket in its place with two hands. The caregiver was also trained to look in front of himself during the whole experiment to avoid influencing the horse with unintentional body language. Then, the horse was led to position (Figure 2; bucket is behind the horse, horse is facing the handler). Then, the handler remained in position without movement and let the horse move freely. The horse approached the food and bent down to eat for 60 s. When a horse bends down for food, it always supports itself with one limb, which is ahead of the other front one (foreleg). We recorded the forward limb as the preferred side. We also recorded the turning side (side preference) from the handler and the bucket each time. After a 60 s eating period, the bucket was removed from the paddock. After an additional 180 s we repeated the process, starting from the original location (Figure 2), in total 10 times. For the temperament analyses (anxiety, trainability and affability), the applied methods were described and validated by Momozowa et al. [25]. This 20-question temperament survey was used for the present study. For each 20-question item, a translation of the original description was given. It was completed by two caretakers (the handler and the trainer) who took care of the horses on a daily basis for at least six months. They scored the horses based on their behavioral traits on a scale of 1 to 9 respectively—1 being the least typical, 9 being the most typical (Supplementary Table S1)—and were not informed in advance of the purpose of the questionnaire, that is, to investigate trait connections to hair whorl characteristics. The similarity score of the answers ranged from 0.84 to 1 according to the Pearson correlation test. The overall score of each trait cluster (anxiety, trainability, and affability) was calculated with the method described by Momozawa et al. [25]. For the summary and the detailed list of the questions, see Section 2.3 and Supplementary Table S1, respectively.

### 2.3. Statistical Analysis

We carried out a frequency calculation for both the position (left, right, center and on the head) and number within the body location (head, body, neck, torso, flank, legs) of hair whorls (circular and linear).

To test whether the laterality index [26] of the animals is different or not, in case of 0.8 cutoff value (which is higher than 0.6 for right and lower than −0.6 for left-handed animals), *t*-test was used. The result was positive; laterality index of the right- and left-handed animals has significantly differed at *p* < 0.05 level. We categorized horses as being left-handed if during the paddock test they used 8 or more times out of 10 their left front leg (or turned left). Horses were considered right-handed if during the paddock test they used 8 or more times out of 10 their right front leg (or turned right). Horses fell into the ambidextrous category if during the paddock test they used 3–7 times out of 10 their left front leg (or turned left).

We carried out a frequency calculation for both the position of the body, neck, torso, flank, limbs) and number within the body location of hair whorls (circular and linear). The presence of circular hair whorls on the upper part of the head, the number of circular hair whorls on the head, which side of the head the circular hair whorl is present (left, right, or ambidextrous), the orientation of the circular head whorls (left, right, or ambidextrous), and the sum of all the hair whorls (both circular and linear) on the head and body were recorded. These variables and the direction of the circular hair whorls on the head (counterclockwise, clockwise or ambidextrous) were compared to leg preference (left, right, or ambidextrous).

We calculated the scores by using the methods described by Momozawa et al. [25,27]. For the list of the questions, see Supplementary Table S1. The number of filled questionnaires was 162. The anxiety cluster contains the following 7 questions:

- Question 1 (Nervousness);
- Question 5 (Excitability);
- Question 9 (Panic);
- Question 11 (Inconsistent emotionality);
- Question 14 (Vigilance);
- Question 18 (Skittishness);
- Question 19 (Timidity).

The trainability cluster contains 4 questions:

- Question 2 (Concentration);
- Question 4 (Trainability);
- Question 8 (Memory);
- Question 15 (Perseverance).

The affability cluster also contains 4 questions:

- Question 6 (Friendliness with people);
- Question 10 (Cooperation);
- Question 13 (Docility);
- Question 16 (Friendliness with horses).

The scores of the questions of each cluster were summed and tested by multivariate general linear model to see if any of the clusters defined above are influenced significantly by the measured variables. Normality was tested by Shapiro–Wilk test. Variables were the presence of a circular hair whorl on the upper part of the head, the number of circular hair whorls on the head, which side of the head the circular hair whorl is present, the direction of the circular head whorls, the sum of all the hair whorls on the horse, on the head, on the body or on the rest of the body, color and leg preference during the paddock test. Tukey post hoc test was used to test the effect of total hair whorl count (either circular or linear hair whorls on the head, body, neck, flack and legs) and the measured personality traits/clusters, respectively.

Pearson correlation test was performed where the post hoc test revealed a significant relationship between the groups and the variables. Calculations were carried out by the SPSS software (version number 29); for significance testing, *p* < 0.05 was used.

## 3. Results

### 3.1. Hair Whorls

Based on our whorl counts (Table 1), circular whorls were more common than linear ones, but their position followed similar patterns. This study examined hair whorls in 81 Hungarian Thoroughbred racehorses. All horses (100%, *n* = 81) had at least one circular whorl on their head or body (neck and flanks). A smaller proportion, 20.9% (17 horses), had circular whorls on their limbs. On the horses’ heads, the median number of circular whorls was 1, with a range from 1 to 3. On the body (excluding the head), the median number of circular whorls was 5, ranging from 2 to 7. Fewer horses (29.6%; 24 horses) had at least one linear whorl on their head. The majority (75.3%, *n* = 61) had linear whorls on their body. Notably, no linear whorls were observed on the limbs of any of the horses. The median number of linear whorls on the head was 0, with a range from 0 to 3. On the body (excluding the head), the median number of linear whorls was 1, ranging from 0 to 5. The median total number of hair whorls (circular and linear) on the head was 2, ranging from 1 to 6. On the body (excluding the head), the median total number of hair whorls was 6, ranging from 2 to 10.

The examined 81 horses had 623 hair whorls on their head in total (circular and linear whorls were 450 and 173, respectively), out of which 70.5% (*n* = 122) were on the upper part of the head, 22.5% (*n* = 39) in between the eyes, and only 12 (6.9%) on the lower part of the head. Out of the 623 hair whorls, 32.7% (*n* = 204) were in the center, 34.0% (*n* = 212) on the left and 32.9% (*n* = 205) on the right side of the head. On the body of the horse (neck, torso and the flank), the 81 examined horses had in total 419 circular and linear hair whorls; 37.2% (*n* = 156) were on the left side, 38.9% (*n* = 163) on the right. On the limbs, the 81 horses had in total 27 circular and 4 linear hair whorls; 51.6% (*n* = 16) were on the left legs and 48% (*n* = 15) on the right legs.

On the head, out of the 120 circular hair whorls, 60 hair whorls were radial (50%), 31 (26.2%) counterclockwise and 26 (21.4%) clockwise. A total of 42 (24.3%) hair whorls were on the left side, 27 (15.6%) were on the right side and 104 (60.1%) were in the center.

On the body (neck, torso and flank), out of the 419 hair whorls, 156 (37.2%), were on the left, and 163 (38.9%) were on the right side. On the leg, out of the 31 whorls, 16 (51.6%) and 15 (48.4%) were on the left and right side, respectively. A total of 27 (87.1%) of them were circular ones.

### 3.2. Paddock Test

Out of 81 horses, 23.5% (*n* = 19) did not have any side preference when turning away from the person who performed the test, 28.4% (*n* = 23) turned more than 80% of the time left, and 48.1% (*n* = 39) turned more than 80% of the time right.

When moving away from the bucket, 53.1% (*n* = 43) did not have any side preference when turning away, 24.7% (*n* = 20) turned more than 80% of the time left, and 22.2% (*n* = 18) turned more than 80% of the time right.

When eating from the bucket, 42.0% (*n* = 34) did not have any side preference (which leg to put forward). However, 33.3% (*n* = 27) had left front leg preference more than 80% of the time, and 24.7% (*n* = 20) had right front leg preference more than 80% of the time.

**Figure 3:**
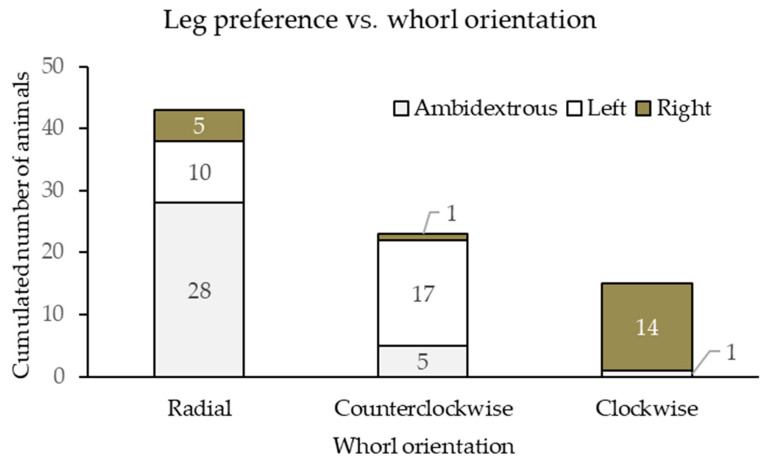
The number of horses showing ambidextrous, left or right leg preference (light gray, white and dark gray boxes, respectively, where the numbers refer to the number of animals with the given categories) during the paddock test and the orientation of the circular head hair whorls (radial, counterclockwise, and clockwise).

There was a significant association between the orientation of the circular hair whorls on the head and the leg preference during the time when horses were eating from the bucket; the Pearson correlation coefficient (r = 0.388, *p* < 0.01) was weak. Out of the 34 horses that did not have any leg preference (ambidextrous), 82.4% (*n* = 28) had either only radial whorls or both clockwise and counterclockwise on their head. Out of the 27 horses that preferred putting their left front leg in forward (left-handed), 63.0% (*n* = 17) had counterclockwise head whorls (or counterclockwise and radial but no clockwise). Out of the 20 horses that preferred putting their right front leg in forward as a foreleg (right-handed), 70.0% (*n* = 14) had clockwise (or clockwise and radial but no counterclockwise) head whorls (Figure 3).

### 3.3. Temperament and Other Parameters

None of the measured parameters on the body or on the head showed significant effect on any of temperament traits (*p* > 0.05 in all cases).

## 4. Discussion

Every animal has its own unique hair whorl pattern that will permanently remain with them during their lifetime; therefore, it is also used as a form of (phenotypic) identification together with other data including DNA information [19]. While professionals usually record only the major whorls on horses, we documented all of the whorls on the body, in detail. All of the eighty-one examined individuals had at least one circular whorl on the head (100%); in contrast, only few animals had it on their limbs (4.3%). This is in line with the findings of Encina et al. [16] where it was 95% and 16% in Pura Raza Español (PRE) horses, respectively. Recent studies show that counterclockwise circular whorls are more common in PRE horses [16] than in Japanese Thoroughbreds [28], and circular whorls are mainly located on the left side of the head, under the central line of the eyes [16]. In our study, most horses showed radial–circular whorls and their presence was much more widespread on the top of the head which is in accordance with the findings of Yokomori et al. [28]. In addition, in our study circular whorls are more common than the linear ones. However, previous studies do not separate the two groups (linear or circular whorls) or do not clarify whether linear and circular whorls are counted together or separately [19,29]. With regard to the total number of hair whorls, our examined horses had more whorls in total and more whorls on the top of the head than the average reported in other studies [16,22,30]. Most often, only one whorl (*n* = 55, 67.9%) occurred on the head, just like in the case of the study of other authors [17,22]. On the other hand, the presence of linear whorls on the head was 30.6% (*n* = 53) in our samples, and the presence of linear whorl across the body including the head and other body parts was 27.8% (*n* = 173). In our observations it was found that whorls on the right side of the body were most likely clockwise, whorls on the left side of the body are usually counterclockwise and whorls on the neck are most commonly radials. The genetic mechanism behind the formation of whorls is unclear, and we do not have accurate scientific information about how their formation is determined by environmental factors [31]. Correlations of whorl direction with anxiety, trainability, and affability were not found (*p* > 0.05). The questionnaire [25], used for scoring temperament traits, was designed to be filled out by those who are familiar with the horses; it would be advisable to check the similarity of the answers produced by non-familiar trainers, as well.

In racehorses, motor lateralization may have influence on athletic performance through turning ability (certain horses become slower in a bend) and speed [32]. For example, having the skill to understand innate motor lateralization should help professionals choose the right training program or racecourse (races are organized at racecourses in clockwise or counterclockwise directions). Furthermore, they have the ability to apply new training methods in order to improve the individual’s balance or to retrain a Thoroughbred that struggles to perform in higher levels due to motor bias.

According to McGreevy and Thomson [26], motor laterality becomes more extreme with age. It was noted [20] that Thoroughbreds older than 2 years were significantly more lateralized than yearlings, and both age groups had more left leg preference than right leg preference. In our study, Thoroughbreds regardless of their age preferred to use the foreleg that was in line with the direction of the whorl on the head (e.g., horses with clockwise whorl preferred to use their right foreleg). This results aligns with previous observations in horses [19,30] and dogs [10]. This is an important association since the heritability (h^2^) of hair whorls are high [17], so theoretically the side preference can also be inherited but the correlation found in this study is considered weak. Along this line, breeders would be able to breed a horse whose motor laterality meets the current needs (e.g., the only racecourse in Hungary has clockwise direction, therefore owners would like to have right-handed or ambidextrous horses). Unfortunately, until now, there is no study that examines the heritability of motor laterality, despite the fact that several authors have long ago called for further studies on this area [24,33].

During the process of creating different horse breeds, artificial selection ensured the fixation of beneficial traits. In case of Thoroughbreds, individuals with excellent speed at the so-called ‘matches’ were the most suitable for further breeding. These horses were usually more competitive and more ‘spirited’ (temperamental) than their counterparts [1]. Nowadays Thoroughbreds are considered as anxious, excitable horses and they are indeed significantly more nervous compared to other breeds [34]. Generally, the other (unofficial) selection factor was the coat color, where showy, nice-looking horses had an advantage [35]. According to popular belief, chestnut-colored horses are bolder and emotionally more inconsistent than others. Finn et al. [36] documented that chestnut horses were bolder than bay-coated horses, and they were more likely to approach novel objects. In our study we did not find correlation between coat color and behavior.

## 5. Conclusions

In Thoroughbreds, circular hair whorls are more dominant than linear ones in all parts of the body; however, most horses had at least one linear whorl in total. Majority of the horses had circular whorls on the top of the head. We found no correlation between reported affability and trainability and total hair whorls on the body. The association between the rotation of the whorls on the head and motor laterality has been proven.

In the future, it would be conducive to map the genetic background of the development of hair whorls in order to find associations between involved genes and the recorded traits.

## Figures and Tables

**Table 1:** Number and percentage of hair whorls present in each location in Hungarian Thoroughbred racehorses. The percentage values represent the proportion of whorl numbers relative to a base quantity of 81 animals.

Location		Without	Top Right	Top Center	Top Left	Between Eyes	Low Right	Low Center	Low Left
Head	Circular	0	13	43	30	22	2	5	5
		0.00	16.05	53.09	37.04	27.16	2.47	6.17	6.17
	Linear	45	12	17	7	17	0	0	0
		55.56	14.81	20.99	8.64	20.99	0.00	0.00	0.00
Body, neck, torso		Without	Right	Center	Left				
	Circular	0	81	51	81				
		0.00	100.00	62.96	100.00				
	Linear	21	27	49	19				
		25.93	33.33	60.49	23.46				
Flank		Without	Right	Left					
	Circular	5	44	46					
		6.17	54.32	56.79					
	Linear	60	11	10					
		74.07	13.58	12.35					
Limbs		Without	Right forelimb	Left forelimb	Right hindlimb	Left hindlimb			
	Circular	32	12	13	1	1			
		39.51	14.81	16.05	1.23				
	Linear	80	2	2	0	0			
		98.77	2.47	2.47	0.00	0.00			

Numbers with two decimal places represent percentage values; above them are the whorl numbers at a given category.

## Data Availability

The original contributions presented in this study are included in the article and Supplementary Materials. Further inquiries can be directed to the corresponding author.

## Supplementary Materials

The following supporting information can be downloaded at https://www.mdpi.com/article/10.3390/vetsci13030289/s1, Table S1: Questionnaire taken from Momozawa et al. [25].

## References

[1] Tolley E.A., Notter D.R., Marlowe T.J. (1985). A review of the inheritance of racing performance in horses. Anim. Breed. Abst..

[2] Lee K.E., Kim J.G., Lee H., Kim B.S. (2021). Behavioral and cardiac responses in mature horses exposed to a novel object. J. Anim. Sci. Technol..

[3] Momozawa Y., Takeuchi Y., Kusunose R., Kikusui T., Mori Y. (2005). Association between equine temperament and polymorphisms in dopamine D4 receptor gene. Mamm. Genome.

[4] Furdon S.A., Clark D.A. (2003). Scalp hair characteristics in the newborn infant. Adv. Neonatal Care.

[5] Grandin T., Deesing M.J., Struthers J.J., Swinker A.M. (1995). Cattle with hair whorl patterns above eyes are more behaviorally agitated during restraint. Appl. Anim. Behav. Sci..

[6] Randle H.D. (1998). Facial hair whorl position and temperament in cattle. Appl. Anim. Behav. Sci..

[7] Meola M.G., Grandin T., Burns P., Deesing M.J. (2004). Hair whorl patterns on the bovine forehead may be related to breeding soundness measures. Theriogenology.

[8] Tomkins L.M., McGreevy P.D. (2010). Hair whorls in the dog (*Canis familiaris*). I. distribution. Anat. Rec..

[9] Tomkins L.M., McGreevy P.D. (2010). Hair whorls in the dog (*Canis familiaris*), Part II: Asymmetries. Anat. Rec..

[10] Tomkins L.M., Williams K.A., Thomson P.C., McGreevy P.D. (2012). Lateralization in the domestic dog (*Canis familiaris*): Relationships between structural, motor, and sensory laterality. J. Vet. Behav..

[11] Smith D.W., Gong B.T. (1973). Scalp hair patterning as a clue to early fetal brain development. J. Pediatr..

[12] Klar A.J. (2003). Human handedness and scalp hair-whorl direction develop from a common genetic mechanism. Genetics.

[13] Weber B., Hoppe C., Faber J., Axmacher N., Fliessbach K., Mormann F., Weis S., Ruhlmann J., Elger C.E., Fernández G. (2006). Association between scalp hair-whorl direction and hemispheric language dominance. Neuroimage.

[14] Cetkin M., Bayko S., Kutoglu T. (2020). Hair Whorl Direction: The Association with Handedness, Footedness, and Eyedness. Dev. Neuropsychol..

[15] Jansen A., Lohmann H., Scharfe S., Sehlmeyer C., Deppe M., Knecht S. (2007). The association between scalp hair-whorl direction, handedness and hemispheric language dominance: Is there a common genetic basis of lateralization?. Neuroimage.

[16] Encina A., Ligero M., Sanchez-Guerrero M.J., Rodriguez-Sainz de Los Terreros A., Bartolome E., Valera M. (2023). Phenotypic and Genetic Study of the Presence of Hair Whorls in Pura Raza Espanol Horses. Animals.

[17] Górecka A., Golonka M., Chruszczewski M., Jezierski T. (2007). A note on behaviour and heart rate in horses differing in facial hair whorl. Appl. Anim. Behav. Sci..

[18] Randle H.D., Webb T.G., Gill L.J. (2003). The relationship between facial hair whorls and temperament in Lundy ponies. Annu. Rep. Lundy Field Soc..

[19] Murphy J., Arkins S. (2008). Facial hair whorls (trichoglyphs) and the incidence of motor laterality in the horse. Behav. Process..

[20] McGreevy P.D., Rogers L.J. (2005). Motor and sensory laterality in thoroughbred horses. Appl. Anim. Behav. Sci..

[21] Krueger K., Schwarz S., Marr I., Farmer K. (2022). Laterality in Horse Training: Psychological and Physical Balance and Coordination and Strength Rather Than Straightness. Animals.

[22] Lima D.F.P.d.A., da Cruz V.A.R., Pereira G.L., Curi R.A., Costa R.B., de Camargo G.M.F. (2021). Genomic regions associated with the position and number of hair whorls in horses. Animals.

[23] Dalin G., Magnusson L.-E., Thafvelin B.C. (1985). Retrospective study of hindquarter asymmetry in Standardbred Trotters and its correlation with performance. Equine Vet. J..

[24] Drevemo S., Fredricson I., Hjerten G., McMiken D. (1987). Early development of gait asymmetries in trotting standardbred colts. Equine Vet. J..

[25] Momozawa Y., Kusunose R., Kikusui T., Takeuchi Y., Mori Y. (2005). Assessment of equine temperament questionnaire by comparing factor structure between two separate surveys. Appl. Anim. Behav. Sci..

[26] McGreevy P.D., Thomson P.C. (2006). Differences in motor laterality between breeds of performance horse. Appl. Anim. Behav. Sci..

[27] Momozawa Y., Terada M., Sato F., Kikusui T., Takeuchi Y., Kusunose R., Mori Y. (2007). Assessing equine anxiety-related parameters using an isolation test in combination with a questionnaire survey. J. Vet. Med. Sci..

[28] Yokomori T., Tozaki T., Mita H., Miyake T., Kakoi H., Kobayashi Y., Kusano K., Itou T. (2019). Heritability estimates of the position and number of facial hair whorls in Thoroughbred horses. BMC Res. Notes.

[29] Abdel-Azeem N.M., Emeash H.H. (2021). Relationship of horse temperament with breed, age, sex, and body characteristics: A questionnaire-based study. Beni Suef Univ. J. Basic Appl. Sci..

[30] Shivley C., Grandin T., Deesing M. (2016). Behavioral Laterality and Facial Hair Whorls in Horses. J. Equine Vet. Sci..

[31] Willems M., Hennocq Q., de Lara S.T., Kogane N., Fleury V., Rayssiguier R., Santander J.J.C., Requena R., Stirnemann J., Khonsari R.H. (2024). Genetic determinism and hemispheric influence in hair whorl formation. J. Stomatol. Oral Maxillofac. Surg..

[32] Warren-Smith A., McGreevy P. (2010). The use of pedometers to estimate motor laterality in grazing horses. J. Vet. Behav..

[33] Grzimek B. (1968). On the Psychology of the Horse.

[34] Sackman J.E., Houpt K.A. (2019). Equine Personality: Association With Breed, Use, and Husbandry Factors. J. Equine Vet. Sci..

[35] Avila F., Hughes S.S., Magdesian K.G., Penedo M.C.T., Bellone R.R. (2022). Breed Distribution and Allele Frequencies of Base Coat Color, Dilution, and White Patterning Variants across 28 Horse Breeds. Genes.

[36] Finn J.L., Haase B., Willet C.E., van Rooy D., Chew T., Wade C.M., Hamilton N.A., Velie B.D. (2015). The relationship between coat colour phenotype and equine behaviour: A pilot study. Appl. Anim. Behav. Sci..

