# Supplementary material for: Hair Whorl Patterns Relating to Equine Behavior and Laterality in Hungarian Thoroughbred Racehorses
Source: Vet Sci. 2026 Mar 19;13(3):289. doi: 10.3390/vetsci13030289 (PMC13030200; doi:10.3390/vetsci13030289)
Supplement: Supplementary file 1 [file vetsci-13-00289-s001.zip › vetsci-4176973-supplementary.pdf]

Supplementary Materials: Hair Whorl Patterns Relating to Equine Behavior and Laterality in Hungarian Thoroughbred Racehorses

Attila Zsolnai, Judit Kis, Boglárka Czinege, László Rózsa, Péter Póti, Ferenc Husvéth and István Anton

Supplementary Table S1. Questionnaire taken from Momozawa et al., [25].

Question number	Items	Description (This horse tends to . . .)	1 - 9
1	Nervousness	become nervous about insects, noises, etc.	Calm - Nervous
2	Concentration	be trainable and undisturbed by the environment	Poor - Excellent
3	Self-reliance	be at ease if left alone away from the herd	Restless - At ease
4	Trainability	be trained easily and promptly	Poor Excellent
5	Excitability	get excited easily	Not excitable - Excitable
6	Friendliness toward people	be never aggressive or fearful	Unfriendly - Friendly
7	Curiosity	be interested in novel objects and approach them	Rarely - Frequently
8	Memory	memorize what it learned or was trained	Poor - Excellent
9	Panic	get excited to an abnormal extent	Never - Frequently
10	Cooperation	be cooperative with a caretaker when handled	Never - Always
11	Inconsistent emotionality	be unpredictable from day to day	Consistent - Inconsistent
12	Stubbornness	be obstinate once it resists a command	Obedient - Stubborn
13	Docility	be docile in general	Active - Docile
14	Vigilance	be vigilant about surroundings	Never - Always
15	Perseverance	be patient with various stimuli	Impatient - Patient
16	Friendliness toward horses	interact with other horses in a friendly manner	Unfriendly - Friendly
17	Competitiveness	be dominant in antagonistic encounters with other horses	Subordinate - Dominant
18	Skittishness	get surprised easily	Not skittish - Skittish
19	Timidity	be timid in a novel environment	Audacious - Timid
20	Gate entrance	go easily through the starting gate	Rarely - Always
